# Blood levels of PAF are elevated during induction of immune complex mediated enteropathy in the rat

**DOI:** 10.1155/S0962935192000516

**Published:** 1992

**Authors:** K. J. Bloch, B. P. Ng, M. Bloch

**Affiliations:** 1 Department of Medicine Harvard Medical School Massachusetts General Hospital Fruit Street, Bulfinch 422 Boston MA 02114 USA; 2 Clinical Immunology and Allergy Units General Medical Services Massachusetts General Hospital Fruit Street, Bulfinch 422 Boston MA 02114 USA

## Abstract

Intravenous injection into rats of immune complexes (IC) prepared in 5 × antigen excess rapidly induces annular bands of vascular congestion and transmural haemorrhage producing a striped appearance of the small intestine. Indirect evidence suggested a major role for PAF in the induction of lesions. In the present study, we showed that blood and leukocyte levels of PAF were elevated in most rats injected 10 min earlier with sufficient IC to induce lesions of 3+ to 4+ intensity. There was no significant difference in the number of rats with elevated plasma levels of PAF. The possibility that changes in blood PAF levels might be mirrored at sites closer to the lesions was considered. The overall effect of PAF on the small intestine of the rats is to induce stasis of flow; the precise target of PAF in mediating this effect is unknown.


Mediators of Inflammation, 1, 341–345 (1992)

### Blood levels of PAF are elevated during induction of immune complex mediated enteropathy in the rat

K. J. Bloch,[1,2,CA] B. P. Ng[2] and M. Bloch[2]

[1]Department of Medicine, Harvard Medical School; and
[2]Clinical Immunology and Allergy Units, General Medical Services, Massachusetts General Hospital, Fruit Street, Bulfinch 422, Boston, MA 02114, USA

[CA] Corresponding Author

**INTRAVENOUS injection into rats of immune complexes (IC) prepared in 5 × antigen excess rapidly induces annular bands of vascular congestion and transmural haemorrhage producing a striped appearance of the small intestine. Indirect evidence suggested a major role for PAF in the induction of lesions. In the present study, we showed that blood and leukocyte levels of PAF were elevated in most rats injected 10 min earlier with sufficient IC to induce lesions of 3+ to 4+ intensity. There was no significant difference in the number of rats with elevated plasma levels of PAF. The possibility that changes in blood PAF levels might be mirrored at sites closer to the lesions was considered. The overall effect of PAF on the small intestine of the rats is to induce stasis of flow; the precise target of PAF in mediating this effect is unknown.**

**Key words:** Enteropathy; Immune complex, Platelet activating factor, Radioimmunoassay for PAF

### Introduction

Immune complex induced enteropathy refers to lesions of the small intestine which develop within minutes of the intravenous injection of immune complexes (IC) into normal rats.[1] The lesions consist of annular bands of hyperaemia alternating with nonhyperaemic bands, causing a striped appearance. Histologically, mild lesions are accompanied by vascular congestion and mucosal oedema; severe lesions are haemorrhagic throughout the intestinal wall and show epithelial necrosis and sloughing of the tips of villi.[1]

Because platelet activating factor (PAF) appears to be a major mediator of reactions elicited by IC[2] and because lesions induced by PAF share some characteristics with those induced by IC,[3,4] we previously tested the effect of dietary manipulations that limit the availability of PAF[5] and of three different PAF receptor antagonists,[6] on the development of lesions. Rats fed a diet supplemented with fish fat had a significantly lower lesional score compared to those on a beef fat supplemented diet.[5] PAF receptor antagonists were able to completely or partially inhibit IC induced small intestinal lesions suggesting that PAF is a major mediator of IC induced enteropathy. In the present experiments, we utilized a newly developed radioimmunoassay to measure PAF levels in blood, plasma and leukocytes of rats undergoing IC induced intestinal lesions.

### Methods

*Animals:* Male Sprague-Dawley rats weighing 125–250 g were obtained from Taconic Farms, Germantown, New York and were maintained in accordance with the guidelines in NIH publication 85-23. Experimental protocols were approved by the Subcommittee on Animal Care, Committee on Research, Massachusetts General Hospital. Animals were fed rat chow devoid of cow's milk proteins.

*Preparation of antisera and immune complexes:* The preparation of antisera and IC has been described in detail.[1,7] Under ether anaesthesia, rats were injected with BSA emulsified in complete Freund's adjuvant and were twice reinjected with BSA in incomplete Freund's adjuvant. IC were prepared in 5 × antigen excess. The lesions, which developed within 10 min of the injection of IC in all four segments of the small intestine, were scored as reported.[1,6] The composite lesional score representing the sum of the scores of the four segments was determined and divided by four so that the resulting numbers correspond to the conventional 0 to 4+ scoring system. The dose of IC selected for the present study produced lesions of 3+ to 4+ severity.

*Measurement of PAF in blood, plasma and leukocytes:* Ten minutes after the intravenous injection of IC, rats were anaesthetized with ether and blood was

© 1992 Rapid Communications of Oxford Ltd obtained by cardiac puncture. PAF was extracted as specified in the manual accompanying the [$^{125}$I]-PAF RIA kit (NEK-062, Dupont/New England Nuclear); siliconized glassware was used throughout. In brief, 2 ml blood was transferred into a glass tube containing 2 ml 20% acetic acid, and 10 $\mu$l of [$^3$H]-PAF (approx 2000 cpm) was added and the mixture vortexed for several seconds. Thereafter, 4 ml 10% acetic acid was added and the suspension vortexed for 10 s. The mixture was centrifuged at 15 000 × $g$ for 45 min, the supernatent was retained. One ml glacial acetic acid was added to the precipitate and the mixture was vortexed vigorously; 9 ml double distilled $H_2O$ (which was used throughout the assay) was added, the mixture vortexed and centrifuged as above for 30 min. The combined supernatants were applied to 200 mg Bond-Elut columns (Analytichem International, Harbor City, CA, USA) pre-treated by successive washing with methanol, water and 10% acetic acid. After loading, the column was treated three times with ethylacetate to remove acetic acid, lipids and water, and was eluted with methanol. The eluate was combined with $H_2O$ and chloroform in a final ratio of chloroform/methanol/water 1:1:0.9. The suspension was allowed to separate into two phases; the bottom phase (chloroform) was recovered, evaporated to dryness and stored. The powder was reconstituted in 1.0 ml of 50 mM sodium citrate buffer pH 6.3. Thereafter, the sample was treated exactly as specified by the manufacturer of the PAF RIA kit.

To obtain plasma, 2.0 ml blood was drawn into a heparinized syringe, cooled immediately in a test tube at 0°C and promptly centrifuged at 500 × $g$ for 10 min at 4°C. Plasma, 0.5 ml, was mixed with 8.5 ml of cold PBS, and 9.0 ml 20% acetic acid containing 10 $\mu$l [$^3$H]-PAF was added and the mixture vortexed repeatedly for 15 min. The mixture was centrifuged at 500 × $g$ for 15 min, the supernatant was recovered and applied to a Bond-Elut column as described above.

To obtain leukocytes, 2.0 ml heparinized blood was promptly cooled as above, centrifuged at 800 × $g$ for 10 min at 4°C, the buffy coat removed and recentrifuged in a Wintrobe tube at 800 × $g$ for 10 min. The buffy coat was again recovered, washed with 2.0 ml cold PBS and recentrifuged. The pelleted WBC were suspended in 2.0 ml PBS, and 2.0 ml 20% acetic acid was added together with 10 $\mu$l [$^3$H]-PAF and the mixture vortexed. Four millitres of 10% acetic acid was added, mixed and centrifuged at 12 000 × $g$ for 45 min. The supernatant was retained and the precipitate treated as described for blood. The combined supernatants were applied to Bond-Elute columns. Because of the different processing required, blood, plasma and leukocytes were obtained from separate animals.

The PAF RIA is a competitive antigen binding assay in which natural PAF and radiolabelled PAF compete for a limited number of antibody binding sites. The assay was performed as recommended by the manufacturer. The antibody is reported not to react with arachidonic acid, prostaglandin $F_{1\alpha}$, phosphatidylcholine and phosphotidylethanolamine and lyso-PAF.

For results to be accepted, at least 45, 70 and 30% of the [$^3$H]-PAF had to be recovered from each blood, plasma and WBC sample, respectively. The results reported herein were corrected for recovery. In addition, plasma values per ml blood were corrected for change in haematocrit which accompanies injection of IC.[1,2]

*Statistical methods:* Control values were compared by the $\chi^2$ method[8] with those obtained at 10 min and 60 min; 10 min and 60 min values were also compared.

### Results

Blood levels of PAF were measured in controls and in rats injected 10 and 60 min earlier with sufficient IC to induce lesions of 3+ to 4+ intensity. Among rats bled at 10 min after IC, there were significantly more animals with blood levels greater than 2.5 ng per ml compared to either the controls ($p < 0.005$) or animals bled 60 min after IC ($p < 0.01$). There was no significant difference in the number of rats with blood levels greater than 2.5 ng/ml at 60 min compared to the controls (Fig 1).

Although measurement of PAF in plasma showed more animals to have values greater than 2.5 ng/ml at 10 min compared to controls or rats bled 60 min after IC, there was no significant difference in the number of animals with values greater than 2.5 ng/ml among controls, or rats bled at 10 or 60 min after IC (Fig 2).

The concentration of PAF in leukocytes from 1 mL of blood was ≥0.85 ng in one of eight controls, seven of nine rats bled 10 min after IC and three of seven rats bled 60 min after IC. There were significantly more rats with such values at 10 min compared to controls ($p < 0.01$) or rats bled at 60 min after IC ($p < 0.05$). There was no significant difference in the number of animals with ≥0.85 ng PAF at 60 min compared to the control group (Fig 3).

### Discussion

Elucidation of the role of PAF in immune complex-induced injury was greatly facilitated by the introduction of specific PAF receptor antagonists. Studies performed with the PAF receptor

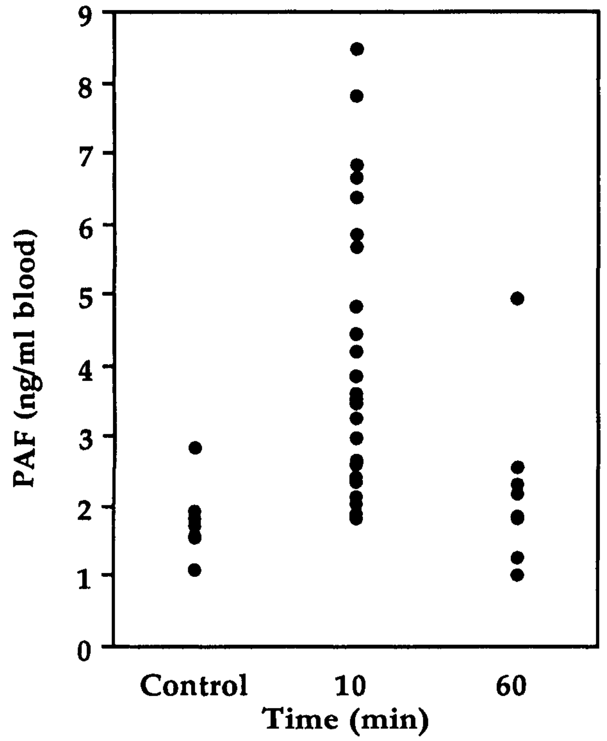

FIG. 1. Blood levels of PAF in control rats and rats injected 10 and 60 min earlier with sufficient immune complexes (IC) to induce lesions of 3+ to 4+ intensity. At 10 min, there were significantly more rats with blood levels >2.5 ng/ml compared to control or rats bled at 60 min.

antagonist L-652,731 by Doebber *et al.* showed that PAF is an important mediator of immune complex induced hypotension and vascular permeability and a minor mediator of immune complex induced lysosomal hydrolase release in the rat. The liver was identified as the probable major site for PAF production in response to administration of IC.[2] Based on studies performed with the same PAF receptor antagonist, Warren *et al.* showed that addition of L-652,731 to the antibody used to induce reverse passive Arthus reactions in the rat, significantly attenuated IC-induced vasculitis.[9] They suggested that PAF contributes to IC induced vascular injury by interacting with PAF receptors on neutrophils.[9] Camussi *et al.* also found that a different PAF receptor antagonist, SRI 63072, inhibited the inflammatory injury induced by the *in situ* formation of IC in the renal glomerulus and skin of the rat.[10] As mentioned above, prior experience with the same experimental model as used herein showed that three different PAF receptor antagonists completely or partially inhibited IC induced enteropathy in the rat.[6] Taken together, these reports strongly link PAF to IC-induced inflammation in the rat.

Limited information is available on changes in blood levels of PAF in response to the administration of IC in any species. In the rabbit, an increase in circulating PAF was detected using the rabbit platelet aggregation test; maximal values were obtained 5–10 min after administration of IC.[11] In the rat, the blood level of PAF in animals subjected to unilateral glomerulonephritis induced by *in situ* formation of IC was 10.5 ± 3.8 ng/ml compared to control values of 2.5 ± 0.8 ng/ml.[12] These levels are much higher than were found in the blood of humans,[12,13] rabbits[11] or dogs.[14] The extent to which the stress of handling and anaesthesia contributes

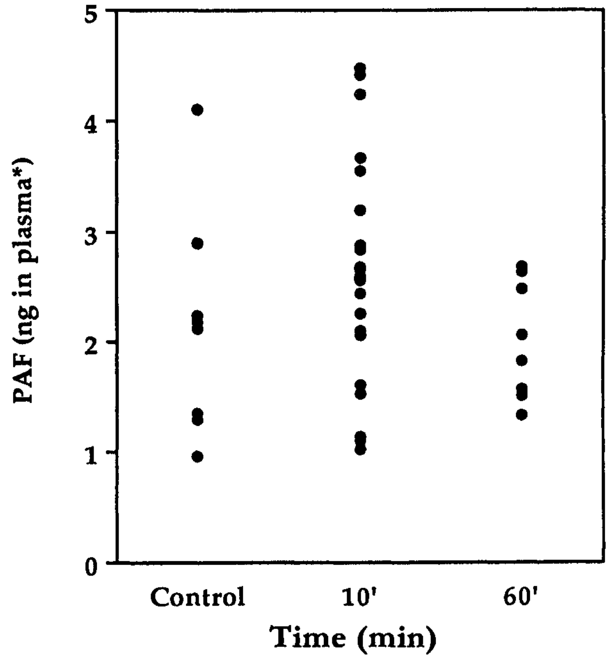

FIG. 2. Plasma levels of PAF in control rats and rats injected 10 and 60 min earlier with IC. There was no significant difference in animals with values greater than 2.5 ng/ml among the three groups. *Values reflect the concentration of PAF in plasma from 1 ml of blood and corrected for the change in haematocrit that follows injection of IC.

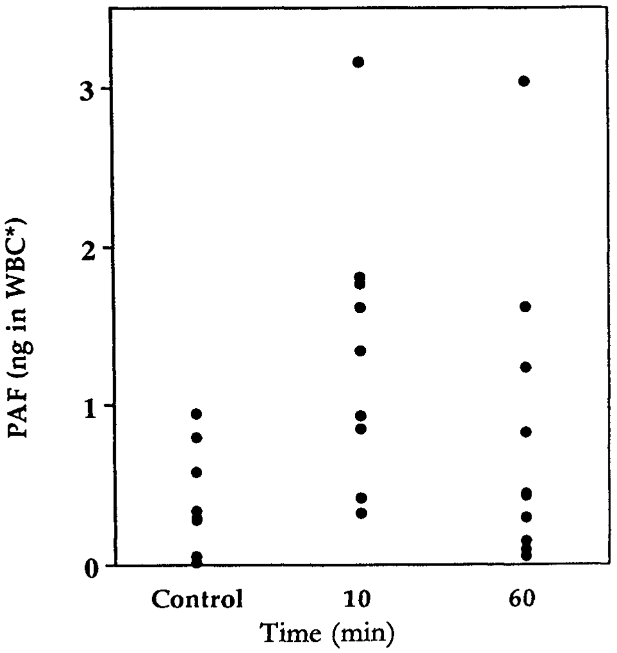

FIG. 3. Concentration of PAF in leukocytes from control rats and rats injected 10 and 60 min earlier with IC. At 10 min, there were significantly more rats with ≥0.85 ng PAF in leukocytes from 1 ml of blood compared to controls or rats bled at 60 min. *Values reflect the concentration of PAF in leukocytes from 1 ml blood.

to the blood levels of PAF found in the rat has not been determined. It is likely that the complexity of bioassays for PAF has hindered more extensive exploration of circulating levels of PAF in spontaneous and experimental inflammatory states. A specific radioimmunoassay for PAF was developed by Sinal *et al.*; it appeared to be at least as sensitive as platelet based assays for PAF, but was simpler to perform and did not have the inherent variability of bioassays.[15] A commercial version of this assay was used in the present study. The blood levels of PAF as measured by RIA in our control rats, approximately 1.8 ng/ml, were comparable to those found by Caramelo *et al.*[12]

It was found that blood and leukocyte levels of PAF were elevated in most rats injected 10 min earlier with sufficient immune complexes prepared in 5 × antigen excess to induce lesions of 3+ to 4+ intensity. There was no significant difference in the number of rats with elevated plasma levels of PAF at 10 min and 60 min after IC compared with the control animals. These findings suggest that the elevated blood values of PAF may reflect the increase in PAF of circulating leukocytes, most likely mononuclear cells, because polymorphonuclear leukocytes disappear almost completely from blood of rats injected 10 min earlier with IC.[2] The findings also provide direct evidence that in IC-induced enteropathy, changes in PAF occur rapidly and do so during the intervals that lesions are developing in the small intestine.

Based on earlier studies suggesting that PAF was involved in the induction of lesions, it had been anticipated that injection of IC might induce a bolus release of PAF into the circulation. Monocytes in the circulation or the fixed mononuclear phagocytes (Kupffer cells) of the liver[2] were considered to be the most likely source of this anticipated bolus release of PAF. Failure to find a marked increase of PAF in plasma under the conditions tested in the present experiments, suggests that the bolus release of PAF may not be involved in the induction of the intestinal lesions provoked by IC, but does not exclude a role for PAF in the induction of lesions.

The plasma levels of PAF may have failed to reflect an increase in PAF in this compartment because it was rapidly cleared by cells bearing PAF receptors including those of the small intestine. Another possibility is that the changes in the blood are paralleled in cells closer to the intestine and that local release of PAF stimulated by IC is responsible for the intestinal lesions. Macrophages in the lamina propria may be one such target for IC stimulation of local PAF release. The local release of PAF in close proximity to the putative target cells or tissues involved in inductions of the intestinal lesions would avoid the modulating effects of plasma proteinase inhibitors on PAF production.[16] Another possibility is that induction of lesions may not require PAF to be released from any cellular source. Changes in intracellular levels of PAF in cells (for example, endothelial cells) at some crucial site might bring about the vascular changes of IC-induced enteropathy.

In earlier studies, it was shown that following the intravenous administration of labelled and 'cold' IC prepared in 5 × antigen excess, radioactivity was found primarily in the liver and small intestine.[7] In the liver, IC have been shown to bind to Kupffer cells, hepatocytes and endothelial cells via Fc receptors.[17] Whether the endothelial cells of rat small intestine have a similar capacity to bind IC and to be stimulated, thereby to increase their synthesis of PAF, remains to be determined. Human endothelial cells have the capacity to synthesize and release PAF in response to a variety of stimuli[18] including the cytokines TNF and IL-1. Although peak release is not achieved until 15 min after stimulation, there is considerable release from endothelial cells by 10 min.[19] Possibly, endothelial cells of the rat small intestine respond similarly.

In the rat intestine, platelet activating factor primarily causes stasis of flow.[20] In severe lesions of IC-induced enteropathy, it appears that inflow of blood into annular segments of the small intestine is less impaired than outflow leading to severe congestion with blood, rupture of vessels and subsequent disruption of the integrity of the villi. The precise target of PAF, whether it is a blood vessel wall structure, nerves regulating vasomotion, or both, is not known.

### References

1. Kirkham SE, Bloch KJ, Perry RP, Walker WA. Immune complex-induced enteropathy in the rat. I. Clinical and histological features. *Dig Dis Sci* 1986; **31**: 737–743.
2. Doebber TW, Wu MS, Biftu T. Platelet-activating factor (PAF) mediation of rat anaphylactic responses to soluble immune complexes. Studies with PAF receptor antagonist L-652,731. *J Immunol* 1986; **136**: 4659–4668.
3. González-Crussi F, Hsueh W. Experimental model of ischemic bowel necrosis. The role of platelet-activating factor and endotoxin. *Am J Pathol* 1983; **112**: 127–135.
4. Wallace JL, Whittle BJR. Profile of gastrointestinal damage induced by platelet-activating factor and endotoxin. *Prostaglandins* 1986; **32**: 137–141.
5. Bloch KJ, Ho B, Xu LL, Bloch M, Robinson DR. Effect of fish-fat or beef-fat supplemented diet on immune complex-induced enteropathy in the rat. *Prostaglandins* 1989; **38**: 385–396.
6. Bloch KJ, Ng BP, Bishara SM, Bloch M. Inhibition of immune complex-induced enteropathy by three different platelet-activating factor receptor antagonists. *Prostaglandins* 1991; **41**: 237–249.
7. Hassell LA, Bishara SM, Bloch KJ. Immune complex-induced enteropathy: effects of repeated injections of immune complexes on the small intestine of the rat. *Am J Pathol* 1989; **134**: 193–201.
8. Zar JH. *Biostatistical Analysis.* Second edition. Prentice-Hall Inc: New Jersey, 1984; 64.
9. Warren JS, Mandel DM, Johnson KJ, Ward PA. Evidence for the role of platelet activating factor in immune complex vasculitis in the rat. *J Clin Invest* 1989; **83**: 669–678.
10. Camussi G, Pawlowski I, Saunders R, Brentjens J, Andres G. Receptor antagonist of platelet-activating factor inhibits inflammatory injury induced by *in situ* formation of immune complexes in renal glomeruli and in the skin. *J Lab Clin Med* 1987: **110**: 196–206.
11. Camussi G, Tetta A, Chiara Deregibus M, Bussolino, F, Segoloni G, Vercellone A. Platelet-activating factor (PAF) in experimentally induced rabbit acute serum sickness: role of basophil derived PAF in immune complex deposition. *J Immunol* 1982: **128**: 86–94.

12. Caramelo C, Fernández-Gallardos, Marin-Cao D, *et al.* Presence of platelet-activating factor in blood from humans and experimental animals. Its absence in anephric individuals. *Biochem Biophys Res Comm* 1984; **120**: 789–796.
13. Chan-Yeung M, Lam S, Chan H, Tse KS, Salari H. The release of platelet-activating factor into plasma during allergen-induced bronchoconstriction. *J Allergy Clin Immunol* 1991; **87**: 667–673.
14. Filep J, Hermán F, Braquet P, Mózes T. Increased levels of platelet-activating factor in blood following intestinal ischemia in the dog. *Biochem Biophys Res Comm* 1989: **158**: 353–359.
15. Sinal MA, Baldo BA, McCaskill A. A specific, sensitive radioimmunoassay for platelet-activating factor (PAF). *J Immunol Methods* 1990; **128**: 183–188.
16. Camussi G, Tetta C, Bussolino F, Baglioni C. Synthesis and release of platelet-activating factor is inhibited by plasma α1- proteinase inhibitor or α1-antichymotrypsin and is stimulated by proteinases. *J Exp Med* 1988; **168**: 1293–1306.
17. Van der Laan-Klamer SM, Harms G, Atmosoerododjo JE, Meijer DKG, Hardonk MJ, Hoedemaeker PJ. Studies on the mechanism of binding and uptake of immune complexes by various cell types of rat liver *in vivo*. *Scand J Immunol* 1986; **23**: 127–133.
18. Bussolini F, Camussi G, Baglioni C. Synthesis and release of platelet-activating factor by human endothelial cells treated with tumor necrosis factor or interleukin 1α. *J Biol Chem* 1988; **263**: 11856–11861.
19. Camussi G, Aglietta M, Malavasi F, *et al.* The release of platelet-activating factor from human endothelial cells in culture. *J Immunol* 1983; **131**: 2397–2403.
20. Mathison R, Rimmer C, Davidson JS, Wallace JL, Befus AD. Alterations in regional blood flow in rats following sensitization to the nematode *Nippostrongylus brasiliensis*: effects of PAF antagonists. *Br J Pharmacol* 1990; **101**: 93–96.

ACKNOWLEDGEMENTS. This study was supported by grant AM 33506 from the National Institutes of Health and by funds from the L. H. Bendit Foundation.

**Received 21 July 1992; accepted 30 July 1992**
